# Adipose-Derived Stem Cells Preincubated with Green Tea EGCG Enhance Pancreatic Tissue Regeneration in Rats with Type 1 Diabetes through ROS/Sirt1 Signaling Regulation

**DOI:** 10.3390/ijms23063165

**Published:** 2022-03-15

**Authors:** Tung-Sheng Chen, Wei-Yu Liao, Chi-Wen Huang, Chin-Hsien Chang

**Affiliations:** 1 Graduate Program of Biotechnology and Pharmaceutical Industries, National Taiwan Normal University, Taipei 106, Taiwan; tschen@ntnu.edu.tw; 2 Traditional Chinese Medicine Department, En Chu Kong Hospital, New Taipei City 237, Taiwan; 01425@km.eck.org.tw (W.-Y.L.); 01644@km.eck.org.tw (C.-W.H.); 3 Department of Cosmetic Science, Chang Gung University of Science and Technology, Taoyuan 333, Taiwan; 4 PhD Program in Clinical Drug Development of Herbal Medicine, College of Pharmacy, Taipei Medical University, Taipei 110, Taiwan; 5 College of Chinese Medicine, China Medical University, Taichung City 404, Taiwan

**Keywords:** green tea EGCG, mesenchymal stem cells, pancreatic regeneration, survival p-Akt

## Abstract

Type 1 diabetes stem-cell-based therapy is one of the best therapeutic approaches for pancreatic damage treatment due to stem cell tissue regeneration. Epigallocatechin gallate (EGCG) is one of the active components found in green tea. Experimental results suggest that EGCG shows beneficial effects on cell protection. This study explores whether a better pancreatic regeneration therapeutic effect could be found in mesenchymal stem cells pretreated with EGCG compared to stem cells without EGCG pretreatment. A cell model confirmed that adipose-derived stem cells (ADSC) incubated with EGCG increase cell viability under high-glucose (HG) stress. This is due to survival marker p-Akt expression. In an animal model, type 1 diabetes induced the activation of several pathological signals, including islet size reduction, extracellular fibrotic collagen deposition, oxidative stress elevation, survival pathway suppression, apoptosis signaling induction, and Sirt1 antioxidant pathway downregulation. Ordinary ADSC transplantation slightly improved the above pathological signals. Further, EGCG-pretreated ADSC transplantation significantly improved the above pathological conditions. Taken together, EGCG-pretreated ADSCs show clinical potential in the treatment of patients with type 1 diabetes through the regeneration of damaged pancreatic tissues.

## 1. Introduction

Clinically, diabetes mellitus (DM) can be identified as a fasting blood glucose over 140 mg/dL and HbA1c (Hemoglobin A1c) over 6.5%. Three types of DM can be clinically recognized, including type 1 DM (insulin secretion reduction due to pancreatic damage), type 2 DM (insulin resistance due mainly to obesity), and gestational DM (developing high blood glucose during pregnancy and usually disappearing after giving birth). Several strategies can be used in the management of DM, including insulin injection, medication, and the maintenance of a good lifestyle [1]. In addition to the above mentioned treatments, stem cell transplantation could potentially be used in type 1 DM treatment due to pancreatic tissue regeneration [2,3].

Reactive oxygen species (ROS) production plays crucial roles in triggering several pathological signals under diabetic conditions. In hyperglycemia, ROS production is mainly related to mitochondrial dysfunction and NADPH activation. Brownleo M. [4] pointed out that respiratory chain overuse in mitochondria under hyperglycemia results in electron transport chain elevation errors, leading to ROS production. On the other hand, hyperglycemia increases advanced glycation end products concentration (AGEs), and AGEs elevation is capable of activating NADPH, resulting in ROS production [5]. ROS overproduction leads to cellular apoptosis. Dehdashtian et al. [6] stated that ROS production induced by hyperglycemia can suppress the formation of mTORC1, which then inactivates autophagy, a process that self-degrades misfolding proteins or damages organelles. Autophagy suppression leads to increased intracellular stress, resulting in programmed cell death. Furthermore, inflammatory response induction by ROS under diabetic status also plays a central role in promoting cell death. Dasu et al. [7] reveals that hyperglycemia increases ROS production, and ROS elevation triggers proinflammatory receptor, TLR4 activation. TLR4 activation expresses downstream proinflammatory cytokines, including IL-1 beta, IL-6, and IL-8, leading to apoptosis. In addition to apoptosis, fibrosis induction is also greatly associated with ROS under hyperglycemia. Proell et al. [8] suggested that ROS induced by hyperglycemia increase TGF beta production. TGF beta expression then activates the fibrotic marker Smad2/3, leading to increased extracellular matrix (ECM). ECM overproduction plays a positive role in tissue fibrosis. Tzouvelekis et al. [9] confirmed that ROS triggers HIF1 alpha expression. HIF1 alpha expression then increases TIMP1 activity and inhibits MMP activities. The imbalance of TIMP1 and MMPs results in ECM production, leading to fibrosis progress.

ROS production is associated with pathological signals under diabetic conditions. Thus, scavenging ROS by antioxidants is essential in ameliorating pathological signals under hyperglycemia. Sirtuin-1 (Sirt 1) acts as one of the deacetylases and functions as a key mediator in antioxidant pathway activation in scavenging ROS under hyperglycemia. Rius-Pere et al. [10] pointed out that Sirt 1 activates PGC-1 alpha expression and PGC-1 alpha expression regulates mitochondrial biogenesis, leading to reduced ROS formation during hyperglycemia. Kobayashi et al. [11] stated that Sirt 1 deacetylates FOX1 and results in antioxidant enzyme expression, including catalase, MnSOD, and thioredoxin, leading to reduced ROS induction by hyperglycemia.

In general, stem cells can be classified as three types, including embryonic stem cells (ESCs), inducible pluripotent stem cells (iPSCs), and mesenchymal stem cells (MSCs). The iPSCs are made from fibroblasts and were first mentioned by Takahashi et al. [12]. The insertion of four key genes (Oct3/4, Sox2, c-Myc, and Klf3) into adult cells enables the transformation of adult cells into cells with pluripotency. Except pluripotency, the insertion of these four genes may increase the risk of tumorigenesis for iPSCs. Thus, the therapeutic effect of iPSCs has mainly been seen in animal studies [13,14]. Although ESCs show a higher pluripotency than MSCs, some disadvantages restrict clinical ESC applications, including ethical issues and tumorigenesis [2,3,12]. Based on the above reasons, MSCs have become the most appropriate therapeutic approaches used in basic and clinical studies. MSC therapeutic tissue regeneration modes can be divided into the paracrine effect and differentiation. Sadat et al. [15] revealed that adipose-derived stem cells (ADSC) release growth factors via the paracrine effect (such as VEGF and IGF-1), and acceptance of these growth factors activates PI3K/Akt survival signaling in damaged cardiomyocytes, leading to cardiac regeneration. Park et al. [16] confirmed that ADSCs secret TGFβ, INFα, and G-CSF, and these factors ameliorate apoptosis signaling in monocytes under deprivation. In addition to the paracrine effect, differentiation is one of the main characteristics for stem cells in performing tissue regeneration. Some papers suggest that direct stem cell contact with cardiomyocytes enables them to stimulate stem cell differentiation into cardiomyocytes, resulting in cardiac regeneration [15,17]. Furthermore, Wang et al. [18] summarized clinical evidence and pointed out that stem-cell-based therapy shows beneficial effects for DM patients. Four hundred ninety-seven DM patients who received mesenchymal stem cells, showed significantly improved pathological factors (including HbA1c, blood glucose level, c-peptide, and insulin requirement) in 3 to 12 months.

Although MSCs show therapeutic effects on diabetes, a stressful environment induced by high glucose is capable of activating pathological signals in MSCs, leading to reduced tissue regeneration capabilities. Ishizuka et al. [19] stated that culturing stem cells in a high-glucose environment will reduce HIF-1α expression and suppress the downstream protein expression, including VEGF and PDGF-B, resulting in tissue regeneration capability reduction. Similarly, a high-glucose environment elevated oxidative damage in stem cells, and elevated oxidative stress increases aging marker p21 expression, leading to stem cell senescence and a reduction in tissue regeneration capability [20,21].

Epigallocatechin gallate (EGCG) is one of the active components found in green tea. Several studies indicated that EGCG exhibits beneficial effects on cell protection, comprising antioxidant, anti-inflammation, anti-apoptosis, and anti-tumorigenesis effects [22,23,24]. Experimental results reveal that culturing stem cells with EGCG increases stem cell capabilities, including increased antioxidant effects, increased differentiation, increased cell proliferation, and increased survival rate under stress [25,26,27]. This study investigates if the pancreatic regeneration capability of MSCs pretreated with green tea EGCG was better than that of MSCs without EGCG pretreatment in diabetic rats receiving MSCs. Cell and animal models were designed to verify the above hypothesis.

## 2. Results

### 2.1. Stemness Characterization for Adipose-Derived Stem Cells

**Figure 1:**
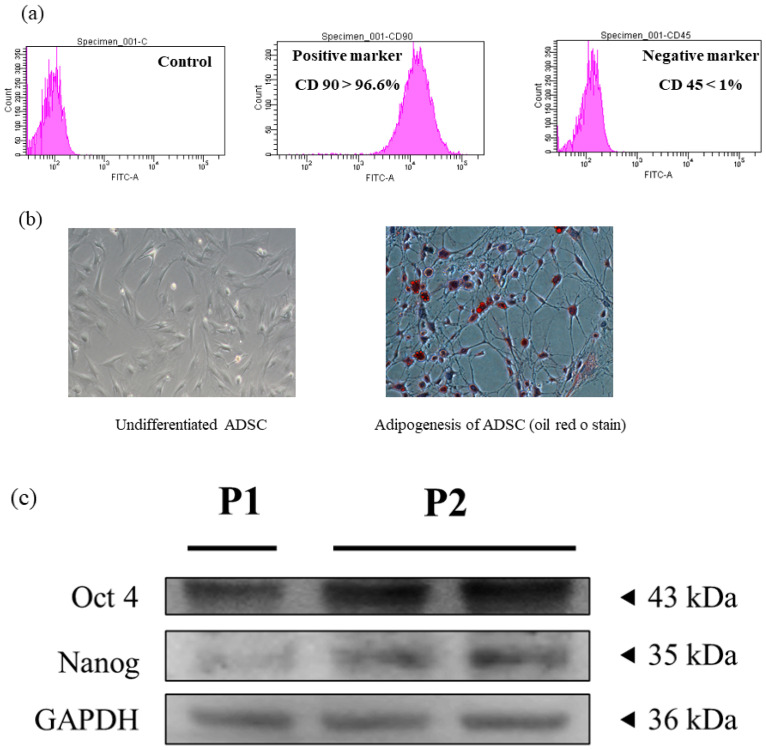
Characterization of adipose-derived stem cells. (**a**) Surface markers, (**b**) adipogenesis differentiation and (**c**) self-renewal markers.

The stemness of experimental cells collected from adipose tissues should be verified before performing experiments. In Figure 1, we see the highly expressed positive marker CD 90 (Figure 1a), the ability for adipogenesis (Figure 1b), and self-renewal markers (Figure 1c) in the experimental cells. Therefore, the experimental cells showed stemness and could be confirmed as adipose-derived stem cells (ADSCs).

### 2.2. Viability and Protein Expression Investigation for ADSCs Cultured with Green Tea EGCG under High-Glucose Stress

**Figure 2:**
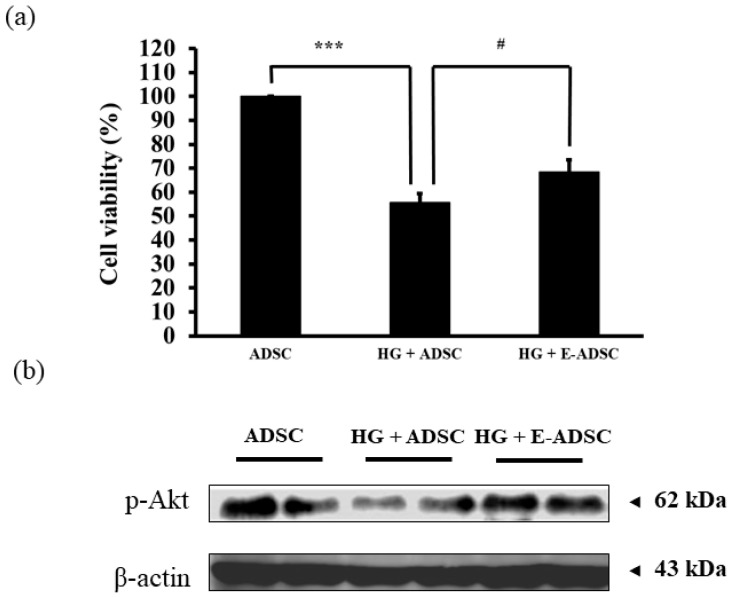
Investigation of survival for ADSC in the presence of high glucose (33 mM). (**a**) Cell viability and (**b**) expression of survival protein marker for ADSC. (*** *p* < 0.001, # *p* < 0.05).

Figure 2 illustrates the viability and protein expression for ADSCs cultured with green tea EGCG in the presence of high-glucose (HG) stress. The viability for ADSC (stem cells alone), HG + ADSC (ADSC with HG) and HG + E-ADSC (EGCG-pre-conditioned ADSC with HG) followed the order 100 ± 6, 56 ± 4 and 68 ± 5%, respectively (Figure 2a), and statistical significance for ADSC > HG + ADSC (*p* < 0.001) as well as HG + ADSC < HG + E-ADSC (*p* < 0.05) was observed. In addition, survival marker p-Akt expression for HG + ADSC was lower than that for ADSC. By contrast, p-Akt expression for HG + E-ADSC was higher than that for HG + ADSC (Figure 2b).

### 2.3. Exploring Serum Glucose, Serum TBARS, and Pancreatic TBARS Levels for Experimental Animals

**Figure 3:**
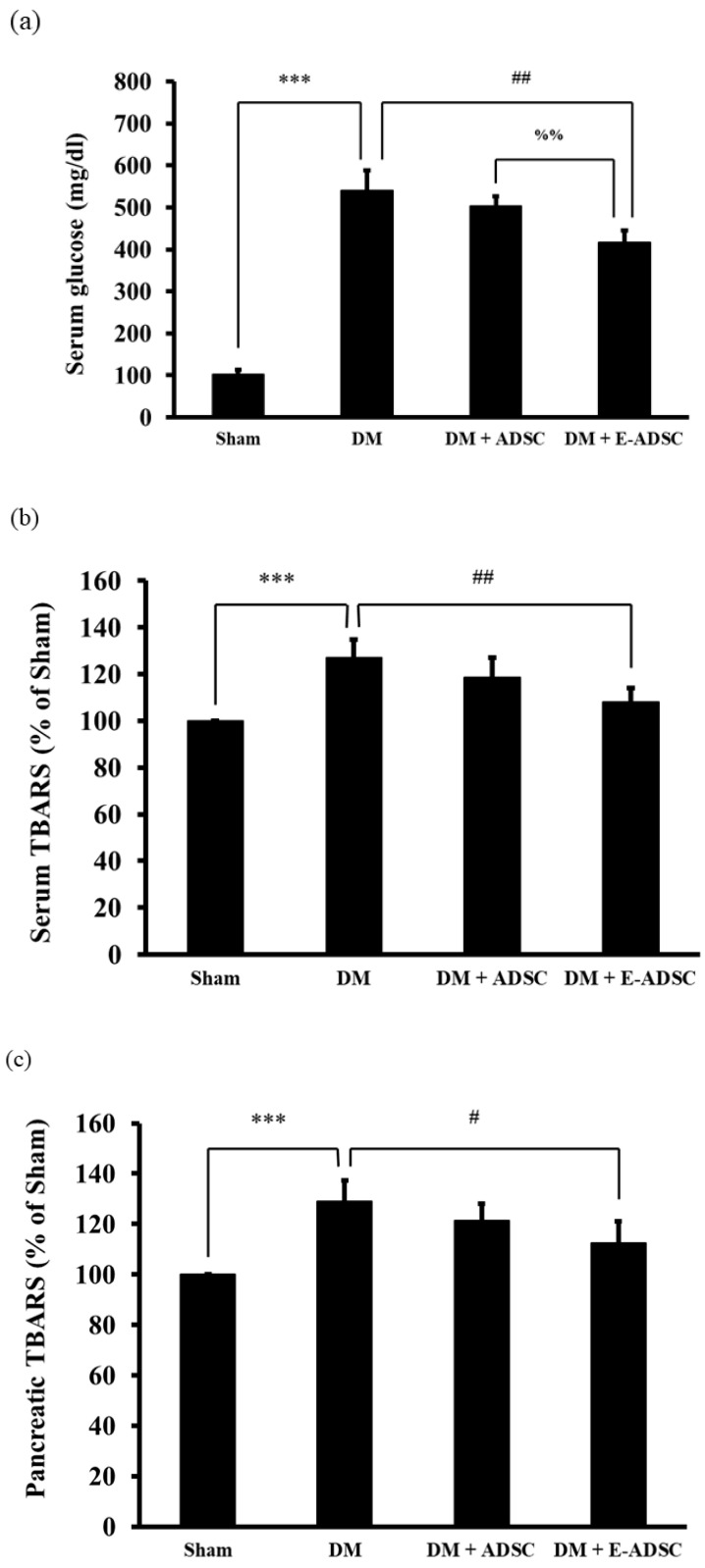
Investigation of (**a**) serum glucose levels, (**b**) serum TBARS levels and (**c**) TBARS pancreatic tissue levels for experimental animals. (*** *p* < 0.001, # *p* < 0.05, ## *p* < 0.01, %% *p* < 0.01).

The serum glucose level was proportional to the pancreatic damage level. In Figure 3a, the serum glucose levels for the sham, DM (diabetic rats), DM + ADSC (diabetic rats with receiving ADSC), and DM + E-ADSC (diabetic rats receiving EGCG-preconditioned ADSC) were 103 ± 10, 540 ± 49, 503 ± 23, and 418 ± 28 mg/dL, respectively. Significance was observed in groups including DM > sham (*p* < 0.001), DM > DM + E-ADSC (*p* < 0.01), and DM + ADSC > DM + E-ADSC (*p* < 0.01). The TBARS level positively correlated with oxidative damage. In Figure 3b, the serum TBARS levels for the sham, DM, DM + ADSC, and DM + E-ADSC were 100 ± 0, 127 ± 8, 119 ± 9, and 108 ± 6% of the sham group, respectively. Significance was observed in groups including DM > sham (*p* < 0.001) and DM > DM + E-ADSC (*p* < 0.01). Similar results were shown for pancreatic TBARS levels. The pancreatic TBARS levels for the sham, DM, DM + ADSC, and DM + E-ADSC were 100 ± 0, 129 ± 8, 121 ± 7, and 113 ± 9% for the sham group, respectively. Significance was observed in groups including DM > sham (*p* < 0.001) and DM > DM + E-ADSC (*p* < 0.05).

### 2.4. Screening Survival Protein Markers for Pancreatic Tissues

**Figure 4:**
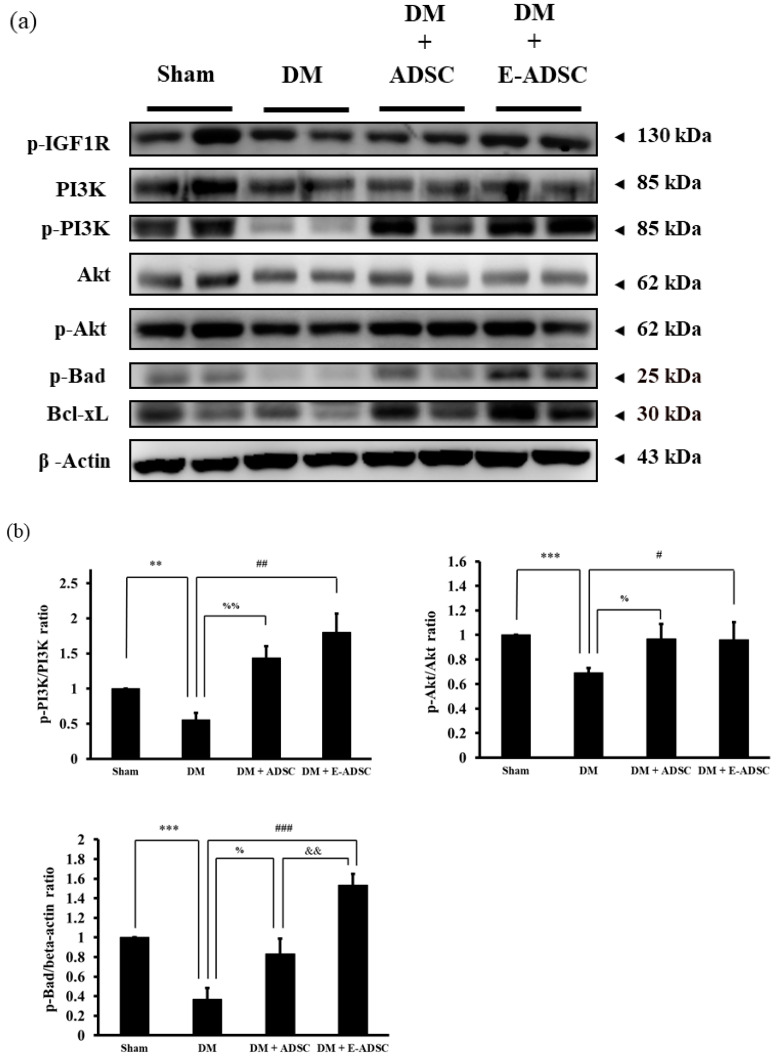
Pancreatic tissue survival protein expression for experimental animals. (**a**) Western blotting analysis and (**b**) quantification. (# *p* < 0.05, ## *p* < 0.01, ### *p* < 0.001, ** *p* < 0.01, *** *p* < 0.001, % *p* < 0.05, %% *p* < 0.01, && *p* < 0.01).

Western blotting analysis was applied to investigate the survival protein expression for pancreatic tissues, including p-IGF1R, PI3K, p-PI3K, Akt, p-Akt, p-Bad, and Bcl-xL. Compared to the sham group, all survival protein markers were suppressed in the DM group. On the other hand, both treatment groups (DM + ADSC and DM + E-ADSC) showed increased survival protein marker expression (Figure 4a). Furthermore, survival marker p-Bad expression in the DM + E-ADSCgroup was significantly higher than that in the DM + ADSC group (DM + E-ADSC > DM + ADSC, *p* < 0.01 shown in Figure 4b).

### 2.5. Screening the Antioxidant Sirt1 Pathway for Pancreatic Tissues

**Figure 5:**
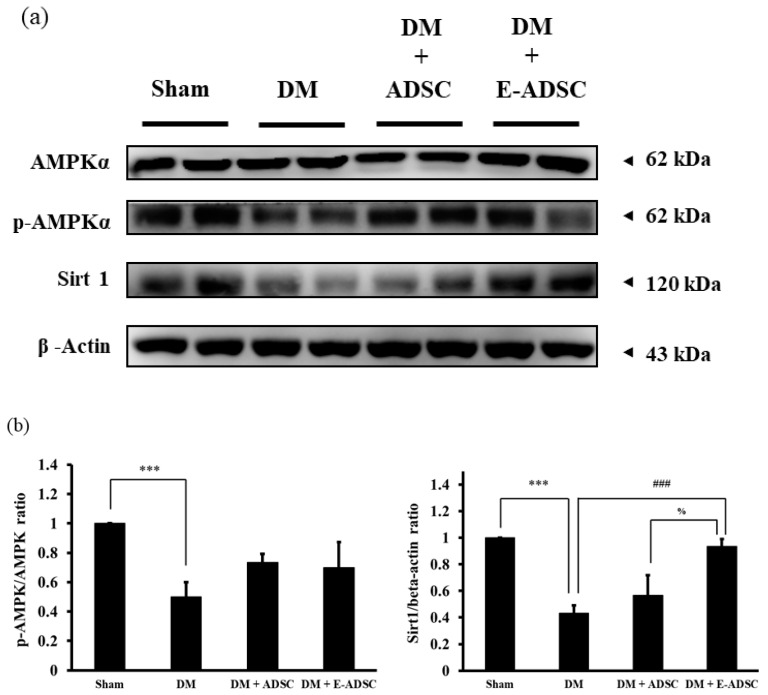
Sirt1 pathway expression for pancreatic tissues in experimental animals. (**a**) Western blotting analysis and (**b**) quantification. (### *p* < 0.001, *** *p* < 0.001, % *p* < 0.05).

The Sirt1 antioxidant pathway, including the AMPK, p-AMPK, and Sirt1 protein markers was investigated in this study. In Figure 5a, we see that p-AMPK and Sirt1 were downregulated in the DM group when compared to the sham group. By contrast, both treatment groups (DM + ADSC and DM + E-ADSC) were capable of increasing p-AMPK and Sirt1 expression, and significant Sirt1 expression was indicated in the DM + E-ADSC group (DM + ADSC < DM + E-ADSC, *p* < 0.05 shown in Figure 5b).

### 2.6. Screening Apoptotic Protein Markers for Pancreatic Tissues

**Figure 6:**
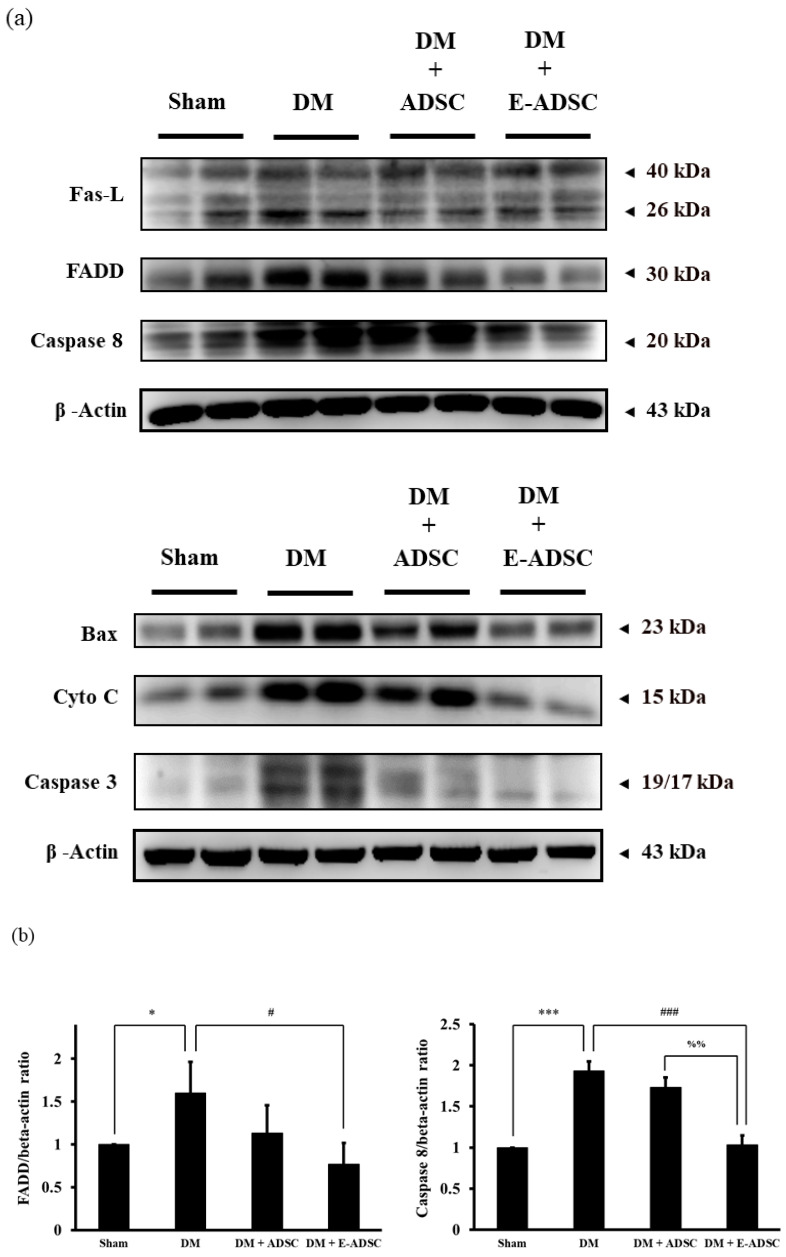

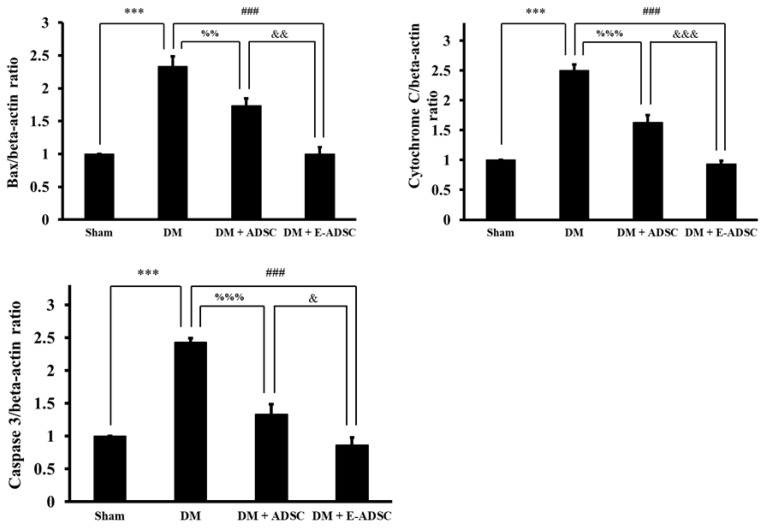
Pro-apoptotic protein expression in pancreatic tissues for experimental animals. (**a**) Western blotting analysis and (**b**) quantification. (# *p* < 0.05, ### *p* < 0.001, * *p* < 0.05, *** *p* < 0.001, %% *p* < 0.01, %%% *p* < 0.001, & *p* < 0.05, && *p* < 0.01, &&& *p* < 0.001).

Apoptotic protein markers, including Fas-L, FADD, caspase 8, Bax, cytochrome C, and caspase 3, were screened in this study. Compared to the sham group, all apoptotic protein markers were upregulated in the DM group. By contrast, the treatment groups (DM + ADSC and DM + E-ADSC) could suppress these apoptotic protein markers (Figure 6a). Among the apoptotic protein markers, FADD, caspase 8, cytochrome C, Bax, and caspase 3 were significantly suppressed in the DM + E-ADSC greater than the DM + ADSC group (shown in Figure 6b).

### 2.7. Investigation of Islet Size for Pancreatic Tissues

**Figure 7:**
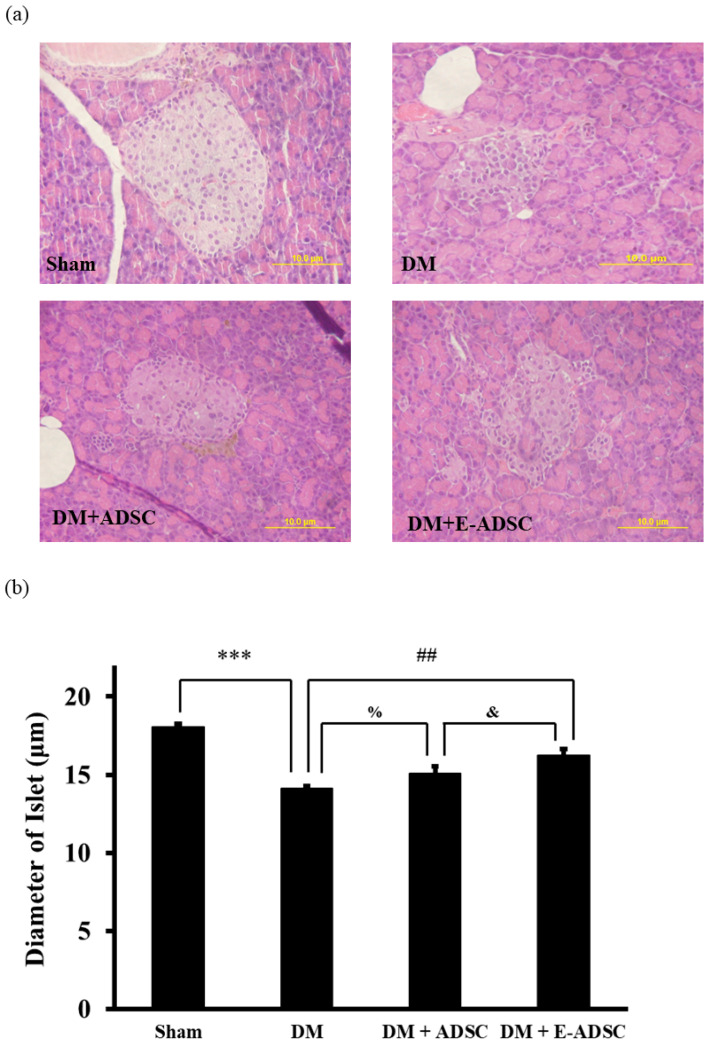
Pancreatic size measurement for experimental animals. (**a**) HE stain analysis and (**b**) quantification. (## *p* < 0.01, *** *p* < 0.001, % *p* < 0.05, & *p* < 0.01).

The islet size for pancreatic tissues was observed using HE staining (Figure 7a). From Figure 7b, we see that the islet sizes for the sham, DM, DM + ADSC, and DM + E-ADSC groups were 18.1 ± 0.2, 14.1 ± 0.2, 15.1 ± 0.5, and 16.2 ± 0.4 µm, respectively. Statistical significance was found in the sham vs. DM (sham > DM, *p* < 0.001), DM + ADSC vs. DM (DM + ADSC > DM, *p* < 0.05), DM + E-ADSC vs. DM (DM + E-ADSC > DM, *p* < 0.01), and DM + E-ADSC vs. DM + ADSC (DM + E-ADSC > DM + ADSC, *p* < 0.05).

### 2.8. Exploring Fibrosis Level for Pancreatic Tissues

**Figure 8:**
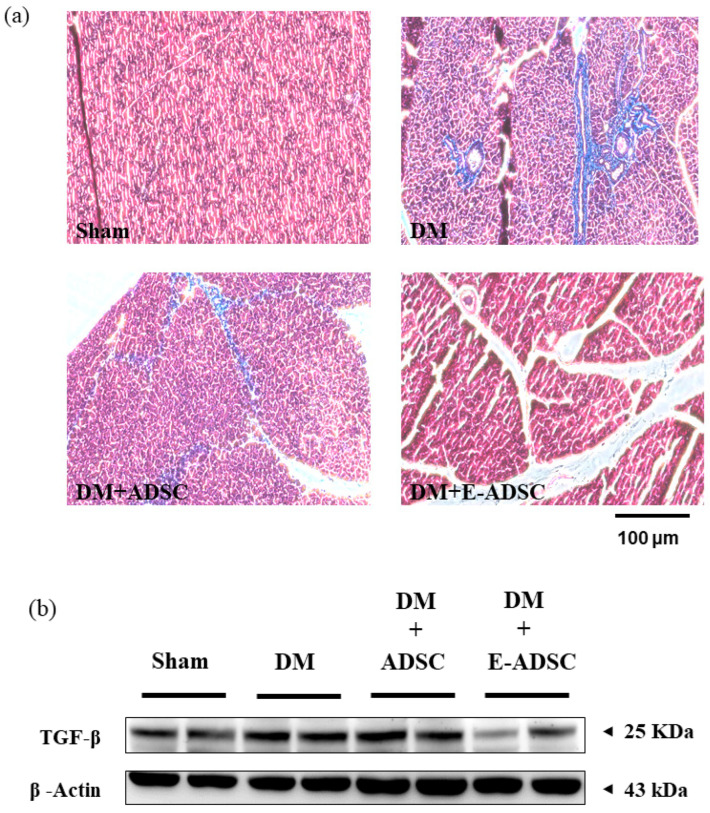
Pancreatic fibrosis analysis for experimental animals. (**a**) Masson’s trichrome stain analysis and (**b**) Western blotting analysis for fibrotic protein markers.

Masson’s trichrome stain was one of the experimental procedures used for collagen deposition visualization in pancreatic tissues. The collagen deposition level was positively associated with the tissue fibrosis level. Compared to the sham group, a large collagen deposition area (blue area) was found in the DM group. By contrast, collagen deposition was improved for both treatment groups (Figure 8a). Furthermore, TGF-beta expression was found in the DM group when compared to the sham group. On the other hand, significant TGF-beta suppression was observed in the DM + E-ADSC group (Figure 8b).

## 3. Discussion

Pancreatic damage is the main causative agent for type 1 diabetes. Thus, pancreatic damage recovery is critical for type 1 diabetes treatment. Stem cell therapy was applied in this study to regenerate the damaged pancreas, leading to a decrease in serum glucose level through pancreatic damage recovery. Before performing experiments, flow cytometry and commercial differentiation kits were applied to confirm the stemness of the experimental cells isolated from adipose tissues (Figure 1a–c). Further, stem cell co-culturing with green tea EGCG confirmed that green tea EGCG is capable of increasing stem cell viability under high-glucose stress through the survival marker p-Akt expression (Figure 2a,b). We needed to confirm if damaged pancreas regeneration by stem cells pretreated with green tea EGCG (called E-ADSC) was better than that from stem cells without green tea EGCG pretreatment (called ADSC).

In order to investigate the therapeutic effect of E-ADSC (EGCG-pretreated ADSC) on pancreatic damage, STZ was applied to animals, and type 1 diabetes was induced through the pancreatic damage induced by STZ. Compared to the sham group, we found that elevated serum glucose level, elevated serum oxidative stress, elevated pancreatic oxidative stress (Figure 3a–c), suppressed survival markers (Figure 4), suppressed antioxidant Sirt1 pathway (Figure 5), expressed apoptotic markers (Figure 6), reduced islet size (Figure 7), and increased fibrotic signaling (Figure 8) were observed in the DM group. On the other hand, both therapeutic groups (including DM + ADSC and DM + E-ADSC) showed improved pathological signals, leading to recovered pancreatic damage and lowered serum glucose level. Furthermore, E-ADSC showed significantly greater improvement in several pathological conditions than ADSC, including lowered serum glucose level (Figure 3a), lowered serum oxidative stress as well as pancreatic oxidative stress (Figure 3b,c), expressed survival marker p-Bad (Figure 4a,b), expressed antioxidant marker Sirt1 (Figure 5a,b), suppressed apoptotic markers caspase 8, Bax, cytochrome C, and caspase 3 (Figure 6a,b), and increased islet size (Figure 7a,b). Therefore, these findings suggest that pretreating green tea EGCG with ADSC shows better therapeutic regeneration effects on the damaged pancreas than ADSC without green tea EGCG pretreatment. 

**Figure 9:**
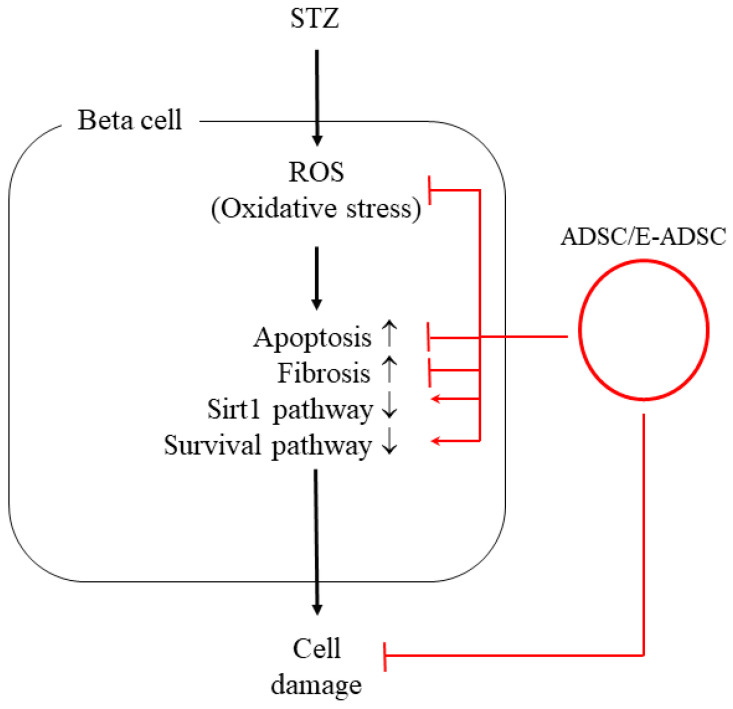
Suggestive signal pathway of this study.

Based on a literature review, we know that elevated oxidative stress can be observed in the diabetic environment, and elevated oxidative stress can trigger various pathological signals in beta cells, including apoptosis and fibrosis. El-Huneidi et al. [28] stated that elevated ROS suppresses the survival marker p-Akt in INS-1 ells (rat beta cell line) under a high-glucose environment, leading to apoptosis in INS-1 cells. By contrast, increased INS-1 cell viability was observed by decreasing ROS through p-Akt upregulation. In addition, Nahdi AMTA et al. [29] stated that streptozotocin (STZ) induces beta cell apoptosis through oxidative stress elevation. Thus, STZ inducing ROS production should be the upstream apoptosis marker activator as well as survival marker suppressor and should be downstream of the cellular signals associated with beta cell damage induced by STZ or the diabetic condition. Furthermore, Roy et al. [30] revealed that ROS triggers TGFβ pathway activation in pancreatic cells, leading to fibrosis in pancreatic tissue in diabetic rats. ROS blockage increases pancreatic cell viability and suppresses the fibrosis status in diabetic rats via TGFβ pathway downregulation. Similar results were found in the following study. Leung PS. [31] indicated that the diabetic condition changes the local renin-angiotensin system (RAS) in the pancreas, and changes in local RAS causes oxidative stress, leading to pancreatic tissue fibrosis. From the above, we conclude that hyperglycemia elevates ROS production (Figure 3), and increased ROS activates pancreatic cell apoptosis (Figure 6) as well as fibrosis formation (Figure 8). These findings are consistent with previous studies, and a suggestive signal pathway is shown in Figure 9.

**Figure 10:**
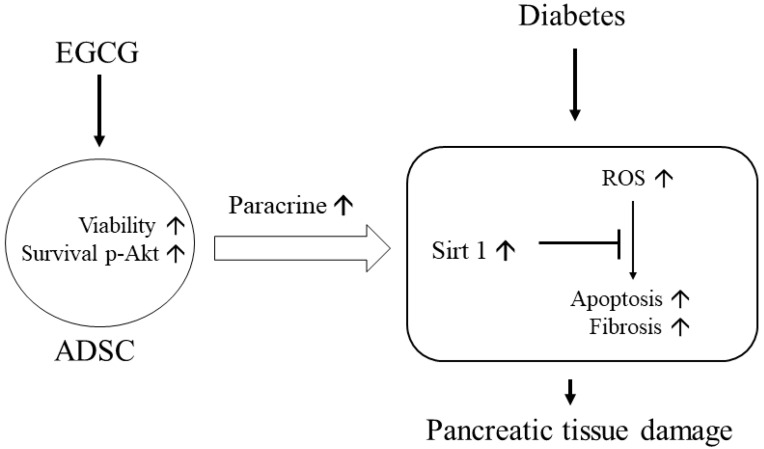
Graphic summary of this study. The upward arrows mean upregulation.

Elevated ROS plays a crucial role in triggering pathological signals in hyperglycemia/diabetic status. Thus, ROS neutralization should be critical in preventing pancreatic tissue damage. Chen et al. [32] pointed out that high glucose causes beta cell damage through ROS production and Sirt1 suppression. Neutralizing ROS by adding antioxidant berberine is capable of expressing Sirt1, leading to beta cell survival. Therefore, this study demonstrates that ROS is the upstream signal and Sirt1 is the downstream signal in high-glucose-induced beta cell damage. From Figure 3c, a significant ROS reduction was found in DM + E-ADSC. Furthermore, Figure 5a illustrates that significant Sirt1 expression upregulation was found in DM + E-ADSC. These findings may imply that E-ADSC transplantation upregulates Sirt1 expression in pancreatic tissue in diabetic rats. Chen et al. [33] stated that ADSC precondition with resveratrol increases stem cell functions, including migration and paracrine effects. Increased ADSC function accelerates cardiac regeneration in diabetic rats with cardiomyopathy through Sirt1/p-Akt axis activation. The above mentioned findings may imply that ADSC is capable of upregulating Sirt1 expression in damaged cardiomyocytes through the paracrine effect. This study speculates that ADSC pretreatment with EGCG increases the stem cell paracrine effect. This increased stem cell paracrine effect can accelerate Sirt1 expression in damaged pancreatic cells. Sirt1 upregulation leads to antioxidant gene expression, such as SOD and HO-1, resulting in neutralizing ROS and pathological signaling blockage, including apoptosis and fibrosis (shown in Figure 10).

In summary, although ADSC preincubation with green tea EGCG shows clinical potential in the treatment of pancreatic tissue damage through increased stem cell paracrine effect and ROS blockage by antioxidant Sirt1 expression, the mediators associated with the paracrine effect and Sirt1 expression should be further studied in the future to elucidate the signaling between stem cells and pancreatic cells.

## 4. Materials and Methods

### 4.1. Chemicals and Reagents

All chemicals and reagents used in this study were purchased from Merck (Merck KGaA, Darmstadt, Germany). The antibodies used in this study were purchased from different manufactures including CD90 and CD45 (BD Biosciences, East Rutherford, NJ, USA); p-Akt, p-IGF1R, PI3K, p-PI3K, Akt, p-Bad, Bcl-xL, Fas-L, and FADD (Cell Signaling Technology, Danvers, MA, USA); b-actin, AMPKa, p-AMPKa, and Sirt1 (Santa Cruz Biotechnology, Dallas, TX, USA); and Caspase-8 and TGF-b (Promega-US).

### 4.2. Isolation and Culture of Adipose-Derived Stem Cells (ADSCs)

The stem cells used in this study were collected from rat adipose tissues. Briefly, epididymal fat tissues were minced and digested with digestion solution containing 0.2% type 2 collagenase (in phosphate-buffered saline) under 37 °C for 3 h. After digestion, the mixture solution was centrifugated and the pellet was collected. The pellet was then resuspended and cultured in a medium containing 10% fetal bovine serum, 2 mM L-glutamine, 100 U/mL penicillin, and 100 ug/mL streptomycin at 37 °C with 5% CO_2_. 

### 4.3. Stem Cell Characterization

The pluripotency of stem cells under passage 1 to 2 used in this study was analyzed using surface markers, self-renewal proteins, and differentiation capability. In this study, CD 90 served as the positive marker and CD 45 served as the negative marker. The surface marker analysis was conducted using flowcytometry (FACSAria III, BD Biosciences, East Rutherford, NJ, USA). The determination of the differentiation capability of stem cells was performed using the mesenchymal adipogenesis kit (Merck KGaA, Darmstadt, Germany) based on the manufacturer’s instructions. Briefly, cells were cultured in adipogenesis medium at less than 37 °C for 21 days. After culturing, cells were stained with Oil Red O staining solution, and the appearance of red cells indicated that the stem cells showed the capability to differentiate into adipocytes.

### 4.4. MTT Cell Viability

The MTT assay determined cell viability. Briefly, cells were cultured in MTT solution under 37 °C for 3 h. After incubation, cells with purple color were formed due to the reaction between the MTT reagent and mitochondria in viable cells. The purple cells were then extracted by adding isopropyl alcohol and the optical density (OD) was read using an ELISA reader with a wavelength of 570 nm. The OD value was proportional to the cell viability.

### 4.5. Animal Model

Male Wistar rats (8 weeks old, 200~250 g) were purchased from BioLASCO (Ilan city, Ilan, Taiwan). The experimental rats were divided into four groups, including sham (without treatment), DM (rats with STZ 50 mg/kg treatment), DM + ADSC (DM rats with transplantation of adipose-derived stem cells, ADSC), DM + E-ADSC (DM rats with transplantation of EGCG-preincubated ADSC, E-ADSC). In the DM, DM + ADSC, and DM + E-ADSC groups, all the diabetic rats were selected in accordance with blood glucose over 200 mg/dL. ADSC or E-ADSC (1 × 10^6^ cells/rat) were dispensed in 0.5 mL of phosphate-buffered saline (PBS) and injected using a 1 mL injection syringe with 27G (1/2” inch) needle via the tail vein for 2 months. All rats were housed at the experimental animal center (China Medical University) with a constant temperature and light cycle (light provided from 7:00~18:00). Food and water were provided ad libitum. The animal model in this study was designed according to the National Institutes of Health Guide for the Care and Use of Laboratory Animals and was approved by the Institutional Animal Care and Use Committee of China Medical University (IACUC-2016–208).

### 4.6. Blood Glucose Determination

Blood samples taken from the animal tail vein were measured for blood glucose level using examination chips (Accu-Chek Active, F. Hoffmann-La Roche Ltd., Little Falls, NJ, USA).

### 4.7. Oxidative Stress for Plasma and Tissue Determination

Thiobarbituric acid (TBA) can react with oxidized lipoproteins or lipids to form TBA-reactive substances (TBARS). Briefly, cell lysates were mixed with TBA solution in vials and incubated at 85 °C for 90 min. After incubation, n-butanol was added to each vial and mixed. The vials were then centrifuged at less than 3000 rpm for 10 min and 150 uL of n-butanol was transferred into a 96-well plate. The optical density (OD) was read using an ELISA reader with a wavelength of 520 nm. The OD values were proportional to the level of oxidative stress.

### 4.8. Western Blotting Analysis

The purpose of Western blotting analysis was to identify and quantify the target protein expression. Briefly, 40 μg of protein samples were transferred into SDS gel and separated under constant voltage (70 V). The SDS gel was then placed onto a polyvinylidene difluoride (PVDF) membrane with a constant voltage to perform the protein transfer. After the protein was transferred, the PVDF membrane was placed in an albumin solution. The membrane was then incubated in a solution containing primary and secondary antibodies. Finally, the protein bands on the membrane were visualized using a fluorescent detector (Fujifilm LAS-3000, GE Healthcare). The protein band intensity was measured using ImageJ software.

### 4.9. HE Stain for Pancreatic Tissues

The tissue morphology was observed using HE staining. Briefly, the protocol for HE-stained tissue slides included dewaxing the slides using xylene, rehydrating the slides using distilled water, staining nuclei using hematoxylin solution, differentiating the nuclei using acid alcohol, staining the background using eosin solution, and dehydrating the slices using xylene. After dehydration, the tissue slides were observed using a microscope. 

### 4.10. Masson’s Trichrome Stain for Pancreatic Tissues

Fibrotic collagen deposition in the tissues was observed as a blue color using Masson’s trichrome stain. Briefly, tissue slides were dehydrated using alcohol, washed using distilled water, stained using Weigert’s working solution, washed using distilled water, stained using Biebrich scarlet-acid fuchsin solution, washed using distilled water, differentiated using phosphomolybdic-phosphotungstic acid solution, incubated in Aailine blue solution, washed using distilled water, incubated in acetic acid solution, and dehydrated using alcohol. After dehydration, the tissue slides were observed using a microscope.

### 4.11. Statistical Analysis

All data were expressed as means (*n* = 3) ± SD (standard deviations). A one-way ANOVA was applied to calculate the significance between groups. Statistical significance was considered at the level of *p* < 0.05.

## Data Availability

All data are available within the article.
